# Warning for unprincipled colorectal endoscopic submucosal dissection: Accurate diagnosis and reasonable treatment strategy

**DOI:** 10.1111/den.12016

**Published:** 2012-12-20

**Authors:** Shinji Tanaka, Motomi Terasaki, Nana Hayashi, Shiro Oka, Kazuaki Chayama

**Affiliations:** 1 Department of Endoscopy, Hiroshima University HospitalHiroshima, Japan; 2 Department of Gastroenterology and Metabolism, Hiroshima University HospitalHiroshima, Japan

**Keywords:** colorectal tumor, endoscopic mucosal resection (EMR), endoscopic submucosal dissection (ESD), laterally spreading tumor (LST)

## Abstract

Piecemeal endoscopic mucosal resection (EMR) is generally indicated for laterally spreading tumors (LST) >2 cm in diameter. However, the segmentation of adenomatous parts does not affect the histopathological diagnosis and completeness of cure. Thus, possible indications for piecemeal EMR are both adenomatous homogenous-type granular-type LST (LST-G) and LST-G as carcinoma in adenoma without segmentalizing the carcinomatous part. Diagnosis of the pit pattern using magnifying endoscopy is essential for determining the correct treatment and setting segmentation borders. In contrast, endoscopic submucosal dissection (ESD) is indicated for lesions requiring endoscopic en bloc excision, as it is difficult to use the snare technique for en bloc excisions such as in non-granular-type LST (LST-NG), especially for the pseudodepressed type, tumors with a type V_I_ pit pattern, shallow invasive submucosal carcinoma, largedepressed tumors and large elevated lesions, which are often malignant (e.g. nodular mixed-type LST-G). Other lesions, such as intramucosal tumor accompanied by submucosal fibrosis, induced by biopsy or peristalsis of the lesion; sporadic localized tumors that occur due to chronic inflammation, including ulcerative colitis; and local residual early carcinoma after endoscopic treatment, are also indications for ESD. In clinical practice, an efficient endoscopic treatment with segregation of ESD from piecemeal EMR should be carried out after a comprehensive evaluation of the completeness of cure, safety, clinical simplicity, and cost–benefit, based on an accurate preoperative diagnosis.

## Introduction

There is an important difference between tumorous lesions in the esophagus or stomach and those in the colorectum, both of which are indications for endoscopic treatment: those in the esophagus or stomach are usually carcinomatous, whereas those in the colorectum are largely benign adenomatous lesions.^1^–^4^ With increasing refinement of endoscopic submucosal dissection (ESD) and improvement in the associated tools and peripheral devices, both of which have enhanced the safety and clinical simplicity of ESD, this technique is becoming more commonly used.^5^ However, colorectal ESD is still technically difficult and more time-consuming and costly than conventional endoscopic mucosal resection (EMR). At the same time, advancement of pit pattern diagnosis using a magnifying endoscope has enabled preoperative diagnosis of adenomas, carcinomas, adenocarcinomas in an adenoma, and carcinoma without adenomatous components and determination of the invasion depth of carcinoma with high precision.^6^ Outcomes of colorectal adenomas that have been treated using piecemeal EMR show that there are many lesions in the adenoma, a benign tumor, that are completely curable using this method.^2^,^7^ It is clear with respect to both clinical simplicity and health economics that the use of ESD for treating benign adenomatous lesions is an overuse of this technique. In the present review, we discuss the outcomes of ESD and EMR based on reports in the literature, discuss an effective colonoscopic approach for colorectal tumors, and summarize the indications for ESD.

## Standard Endoscopic Treatments in Japan Based on JSCCR Guidelines 2010 for the Treatment of Colorectal Cancer

Early-stage colorectal carcinoma, which is unlikely to metastasize to lymph nodes and can be excised en bloc, is usually treated using an endoscopic approach.^8^ In practice, however, because of the limitation of preoperative diagnosis for eliminating precancerous lesions, a number of benign adenomatous lesions are treated using endoscopy. Preoperative discrimination between adenomas, carcinomas in adenoma, and carcinoma without adenomatous components and identification of the histological grade of carcinoma before treatment is extremely important for selection of the appropriate therapy.

**Figure 1:**
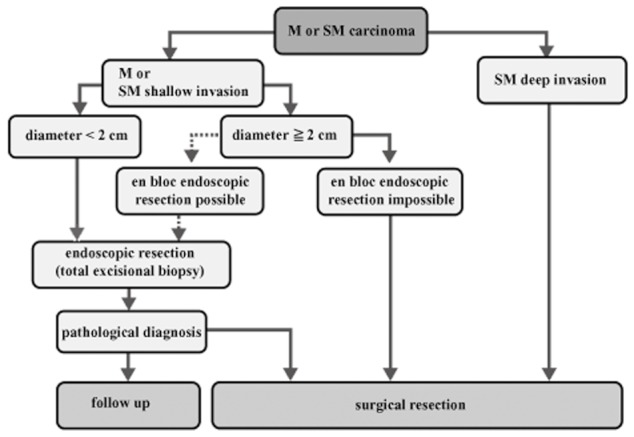
Therapeutic strategy for lesions diagnosed as M or SM carcinoma (JSCCR Guidelines 2010 for Treatment of Colorectal Cancer).

**Figure 2:**
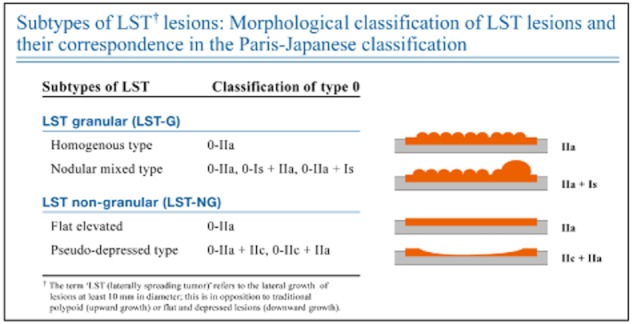
Laterally spreading tumor (LST) and its subclassification in relation to macroscopic type classification.

Based on the Japanese Society for Cancer of the Colon and Rectum (JSCCR) Guidelines 2010 for the Treatment of Colorectal Cancer, the following conditions can be treated endoscopically (Fig. 1):^8^ (i) adenoma/mucosal (M) cancer or shallow invasive carcinoma into the submucosa; (ii) tumor of diameter <2 cm; and (iii) any macroscopic type of tumor. The average diameter of lesions that can be treated with en bloc snare EMR is approximately 2 cm; hence, for a tumor to be treated endoscopically, its maximum diameter should be <2 cm. When ‘carcinoma’ is endoscopically treated, precise histological analysis of the excised specimen is essential, and en bloc excision is a fundamental requirement. In addition, adenomatous lesions and carcinomas are frequently found in the colorectum, as mentioned above. When their clinicopathological characteristics can be well examined before treatment, lesions ≥2 cm in diameter, for which en bloc snare EMR is not indicated, can be completely cured using deliberate piecemeal EMR to avoid segmentation of the carcinomatous part without affecting the pathological diagnosis. Most lesions to which en bloc excision using snare EMR cannot be applied are laterally spreading tumors (LST) (Fig. 2),^9^ and they are defined as superficial spreading-type tumors with a maximum diameter >1 cm. LST can be clinicopathologically characterized in detail before treatment by examining their subtype or pit pattern and can be regarded as a lesion group to which piecemeal EMR is applied relatively often, even if their maximum diameter exceeds 2 cm.

## Indications for Piecemeal EMR in LST

Many adenomatous lesions in the colorectum can be discriminated from carcinomas by applying pit pattern analysis based on magnifying endoscopic examination.^6^,^9^ Generally, lesions with a regular pit pattern, such as type II, III or IV, are intramucosal, and those with type V_N_ (non-structure) pit pattern are deep invasive submucosal (SM) carcinomas. They can be easily diagnosed on routine daily colonoscopic examination. Most large tumors with diameters >2 cm are usually LST and, of them, most granular-type LST (LST-G) are recognized as adenomatous lesions. Among homogenous-type LST-G, the occurrence of carcinoma or SM invasion is extremely rare. In granular-nodular-mixed-type LST-G, SM invasion may exist in large tubercles or as part of a type V_I_ pit pattern. Because LST-G is either an adenoma or a focal carcinoma in adenoma, the carcinomatous portion may form a large nodule and pit pattern diagnosis can be carried out. In addition, deliberate piecemeal EMR without excising the carcinomatous portion in pieces can be applied.^1^,^2^,^10^,^11^ A different approach is required in the case of non-granular-type LST (LST-NG), because these are associated with a higher incidence of carcinoma and SM invasion than LST-G and therefore need to be treated more carefully. In particular, a pseudodepressed type of LST-NG demonstrates a high probability of multifocal SM invasion, irrespective of its size and pit pattern. Therefore, en bloc excision and not piecemeal EMR should be applied, and the specimen obtained from complete en bloc excision should be pathologically diagnosed in detail.^1^,^2^,^10^,^11^

As mentioned above, pit pattern diagnosis based on magnifying endoscopy is very useful for deciding upon indications for ESD or for piecemeal EMR. In addition, magnifying endoscopy using image enhanced endoscopy (IEE) approaches such as narrow band imaging (NBI) and Fuji intelligent color enhancement (FICE), which have prevailed in recent years, also enable detailed characterization of tumorous lesions and are useful for identifying lesions to which piecemeal EMR can be applied.^12^–^16^

Previous reports have shown that when piecemeal EMR was applied to lesions strictly selected on the basis of preoperative diagnosis, almost all of the recurrent lesions were adenomas and additional endoscopic treatment resulted in a complete cure; this approach is thus established as acceptable clinical practice.^17^,^18^ A research project initiated by the JSCCR, ‘Multi-center prospective cohort study on localized complete cure and complications when various endoscopic resection techniques are applied to colorectal tumors with maximum diameter more than 2 cm,’ has been completed.^19^ This project was designed to examine the safety and efficacy of various endoscopic treatments, including piecemeal EMR and ESD, for colorectal tumors with a maximum diameter >2 cm. We are looking forward to the results on localized completeness of cure, which will be reported soon.

## Outcomes of Colorectal EMR in the Literature

The PubMed database was used to search for publications related to colorectal EMR using the key words ‘EMR’ and ‘colon’. The MEDLINE database was used to search for publications through April 2012 related to EMR using the above-mentioned key words. A manual search of the citations of relevant articles was also carried out.

Outcomes of colorectal EMR obtained from the literature review are summarized in Table 1.^7^,^17^,^20^–^43^ Target lesions and technical level of EMR differed greatly between reports; average rate of perforation, rate of postoperative bleeding, and average rate of en bloc resection were 0.7% (0–5.8%, 20/2755), 4.5% (0–16.0%, 124/2755), and 42.6% (19.2–91.8%, 1080/2538), respectively. Rates of local recurrence for en bloc resection and piecemeal resection were 4.0% (0–17.9%, 31/784) and 17.0% (4.8–31.4%, 214/1257), respectively; the latter was significantly higher than the former. Notably, no additional surgical resection was required in 88.4% (40–100%, 221/250) of all cases. Hence, the EMR technique, including piecemeal EMR, may be a useful therapeutic method for overall treatment. Importantly, when preoperative diagnosis was carried out in detail, recurrent lesions were usually found to be adenomas and additional endoscopic treatment was successful in attaining complete cure.

**Table 1:** Outcomes of colorectal endoscopic mucosal resection in the literature

			Resection rate		Complications		Local recurrence rate		Additional treatment	
						
Author	No. cases	Size of lesion (mm)	Endoscopic en bloc resection (%)	Histological complete resection (%)	Perforation (%)	Postoperative bleeding (%)	Endoscopic en bloc resection (%)	Endoscopic piecemeal resection (%)	Endoscopic treatment (%)	Surgery (%)
Kobayashi *et al*.^20^	56	25 ± 9	21/56 (37.5)		0/56 (0)	1/56 (1.8)	1/21 (4.8)	11/35 (31.4)	11/12 (91.7)	1/12 (8.3)
Terasaki *et al*.^7^	178	32	70/178 (39.3)	176/178 (98.9)	3/178 (1.7)	15/178 (8.4)	1/69 (1.5)	13/107 (12.1)	14/14 (100)	0/14 (0)
Santos *et al*.^21^	172	11.5 ± 9.6	158/172 (91.8)	155/172 (90.1)	0/172 (0)	5/172 (2.9)	1/109 (0.9)	4/13 (30.8)	5/5 (100)	0/5 (0)
Sakamoto *et al*.^22^	222	28.2 ± 12.5			0/222 (0)	4/222 (1.8)		42/222 (18.9)	39/42 (92.9)	3/42 (7.1)
Lee *et al*.^23^	140	21.7 ± 3.5	60/140 (42.9)	46/140 (32.8)	0/140 (0)	0/140 (0)			28/29 (96.6)	1/29 (3.4)
Woodward *et al*.^24^	423	20	185/423 (43.7)				9/185 (4.9)	40/234 (17.1)		
Tajika *et al*.^25^	104	25.5 ± 6.8	50/104 (48.1)		0/104 (0)	3/104 (2.9)	1/50 (2.0)	15/54 (27.8)	12/16 (75.0)	3/16 (18.8)
Ferrara *et al*.^26^	182	24.7 ± 10.2	79/177 (44.6)		2/177 (1.1)	22/177 (12.4)	6/78 (7.7)	6/94 (6.4)	9/12 (75.0)	3/12 (25.0)
Seo *et al*.^27^	50	30.1			0/50 (0)	1/50 (2)		5/41 (12.2)	2/5 (40.0)	3/5 (60.0)
Soune *et al*.^28^	26	49			1/26 (3.8)	2/26 (7.7)		3/24 (12.5)	2/3 (66.7)	1/3 (33.3)
Moss *et al*.^29^	80	37.5	18/80 (22.5)	72/80 (90.0)	0/80 (0%)	3/80 (3.8)				
Hochdörffer *et al*.^30^	167	40 ± 14	32/167 (19.2)		3/167 (1.8)	9/167 (5.4)	5/28 (17.9)	21/71 (29.6)	19/26 (73.1)	6/26 (23.1)
Luigiano *et al*.^31^	148	39.7 ± 12.5	65/148 (43.9)		1/148 (0.7)	15/148 (10.1)	2/65 (3.1)	4/83 (4.8)	5/6 (83.3)	1/6 (16.7)
Huang *et al*.^32^	103	32	46/103 (44.7)		0/103 (0)	2/103 (1.9)	1/37 (2.7)	10/42 (23.8)	11/11 (100)	0/11 (0)
Saito *et al*.^33^	228	28 ± 8	74/228 (32.5)		3/228 (1.3)	7/228 (3.1)	2/74 (2.7)	31/154 (20.1)	31/33 (93.9)	2/33 (6.1)
Arezzo *et al*.^34^	27		21/27 (77.8)	27/27 (100)	0/27 (0)	3/27 (11.1)	0/21 (0)	2/6 (33.3)	2/2 (100)	0/2 (0)
									10/10 (100)	0/10 (0)
Mahadeva & Rembacken^35^	224	10			1/224 (0.4)	5/224 (2.2)				
Hurlstone *et al*.^36^	163	9.1	116/163 (71.2)	115/163 (70.6)	1/163 (0.6)	2/163 (1.2)				
Arebi *et al*.^37^	161	32.5			0/120 (0)	2/120 (1.7)				
Katsinelos *et al*.^38^	59	24.4 ± 17.5	23/59 (39.0)		0/59 (0)	4/59 (6.8)			1/2 (50)	1/2 (50)
Bories *et al*.^39^	52	29.8	23/52 (44.2)	51/52 (98.1)	3/52 (5.8)	1/53 (1.9)	2/14 (14.3)	3/19 (15.8)		
Katsinelos *et al*.^40^	21	23.5 ± 13.6	15/21 (71.4)		0/21 (0)	1/21 (4.8)			4/4 (100)	0/4 (0)
Kume *et al*.^41^	30	27	26/30 (86.7)		0/30 (0)	1/30 (3.3)		2/26 (7.7)	2/2 (100)	0/2 (0)
Hurlstone *et al*.^42^	58	38	22/58 (38.0)		0/58 (0)	2/58 (3.4)			8/10 (80)	2/10 (20.0)
Bergmann & Beger^43^	71	25.4	35/71 (49.3)		1/69 (1.5)	1/69 (1.5)	0/33 (0)	2/32 (6.3)		
Tanaka *et al*.^17^	81	31.2 ± 11.0	41/81 (50.6)		1/81 (1.2)	13/81 (16.1)			6/6 (100)	0/6 (0)

## Indications for Colorectal ESD

Colorectal ESD is indicated for lesions that can be managed endoscopically and which require en bloc excision, but would require piecemeal removal if treated with snare EMR. ‘The Colorectal ESD Standardization Implementation Working Group,’ a subordinate organization of the ‘Gastroenterological Endoscopy Promotion Liaison Conference,’ has proposed a draft of ‘Criteria of Indications for Colorectal ESD’ (Table 2).^2^,^5^,^44^ It specifically states that colorectal ESD is indicated for lesions requiring endoscopic en bloc excision, for which it is difficult to use the snare technique, such as LST-NG, especially the pseudodepressed type; tumors with a type V_I_ pit pattern; shallow invasive submucosal carcinoma; large depressed tumors; and large elevated lesions that are probably malignant (large nodular lesions such as LST-G). Other lesions, such as intramucosal tumor accompanied by submucosal fibrosis, induced by biopsy or peristalsis of the lesion; sporadic localized tumors that occur as a result of chronic inflammation, including ulcerative colitis; and local residual early carcinoma after endoscopic excision, are also included in the indications.

**Table 2:** Indications for endoscopic submucosal dissection for colorectal tumors

1 Large sized (>20 mm in diameter) lesions in which en bloc resection using snare EMR is difficult, although it is indicative for endoscopic treatment
LST of the non-granular type (LST-NG), particularly those of the pseudo-depressed type
Lesions showing V_I_ type pit pattern
Carcinoma with submucosal infiltration
Large depressed-type lesion
Large elevated lesion suspected to be carcinoma†
2 Mucosal lesions with fibrosis caused by prolapse due to biopsy or peristalsis of the lesions.
3 Sporadic localized tumors in chronic inflammation such as ulcerative colitis.
4 Local residual early carcinoma after endoscopic resection.

† Including granular-type laterally spreading tumor (LST-G), nodular mixed type by the Colorectal Endoscopic Submucosal Dissection (ESD) Standardization Implementation Working Group.

EMR, endoscopic mucosal resection.

In Japan, colorectal ESD has been covered under health insurance since April 2012. It was decided that its indication would be early malignant colorectal tumors, concretely ‘adenoma and early carcinoma of 2–5 cm in diameter’. The basis for indication is uncertain. The approved indication is inconsistent with the fact that there is no size limitation in the indication for its use in early carcinoma of the esophagus and the stomach. It also fails to reflect the present situation where pit pattern diagnosis based on pit pattern by magnifying endoscopy or magnifying endoscopy using IEE such as NBI/FICE can easily discriminate between adenoma and carcinoma. In fact, obvious adenoma can be radically cured by EMR, including piecemeal resection and, therefore, the use of ESD should be naturally regarded as overtreatment.^7^,^18^,^45^,^46^

## Outcomes of Colorectal ESD in the Literature

The PubMed database was used to search for publications related to colorectal ESD using the key words ‘ESD’ and ‘colon’. The MEDLINE database was used to search for publications through April 2012 related to ESD using the above-mentioned key words. A manual search of the citations of relevant articles was also carried out. Pertinent studies published in English and Japanese were reviewed. If an institution had published several reports on colorectal ESD, the newest report was selected for the summary of outcomes of colorectal ESD.

Outcomes of colorectal ESD using previous reports from single institution studies are shown in Table 3.^47^–^63^ Regarding efficacy, the en bloc resection (endoscopic) and complete en bloc resection (histological) rates were 90.5% (61–98.2%, 2740/3028) and 76.9% (58–95.6%, 1385/1801), respectively. Regarding complications, the perforation and postoperative bleeding rates were 5.4% (1.3–20.4%, 180/3339) and 1.8% (0.5–9.5%, 42/2300), respectively. Local recurrence was detected in 1.9% (0–11%, 20/1036) of cases.

**Table 3:** Summary of outcomes of colorectal ESD using previous reports from single institution studies (non-multicenter study)†

Author	Year	No. cases	Size (mm)	En bloc resection (%)	Complete en bloc resection (%)	Complications		Local recurrence (%)
							
						Perforation (%)	Bleeding (%)	
Tamegai *et al*.^47^	2007	71	32.7	70/71 (98.6)	68/71 (95.8)		1/71 (1.4)	0/71 (0)
Hurlstone *et al*.^48^	2007	42	31	33/42 (78.6)	31/42 (73.8)	1/42 (2.4)	4/42 (9.5)	4/36 (11.1)
Fujishiro *et al*.^49^	2007	200	29.9	183/200 (91.5)	141/200 (70.5)	12/200 (6.0)	1/200 (0.5)	
Zhou *et al*.^50^	2009	74	32.6	69/74 (93.2)	66/74 (89.2)	6/74 (8.1)	1/74 (1.4)	0/74 (0)
Isomoto *et al*.^51^	2009	292	26.8	263/292 (90.1)	233/292 (79.8)	23/292 (7.9)	2/292 (0.7)	1/220 (0.5)
Saito *et al*.^52^	2009	405	40	352/405 (86.9)		14/405 (3.5)	4/405 (1.0)	
Iizuka *et al*.^53^	2009	38	39	23/38 (60.5)	22/38 (57.9)	3/38 (7.9)		
Hotta *et al*.^54^	2010	120	35	112/120 (93.3)	102/200 (51.0)	9/120 (7.5)		
Niimi *et al*.^55^	2010	310	28.9	280/310 (90.3)	231/310 (74.5)	15/310 (4.8)	5/310 (1.6)	4/202 (2.0)
Yoshida *et al*.^56^	2010	250	29.1	217/250 (86.8)	203/250 (81.2)	15/250 (6.0)	6/250 (2.4)	
Toyonaga *et al*.^57^	2010	512	29	503/512 (98.2)		9/512 (1.8)	8/512 (1.6)	
Matsumoto *et al*.^58^	2010	203	32.4		174/203 (85.7)	14/203 (6.9)		
Uraoka *et al*.^59^	2011	202	39.9	185/202 (91.6)		5/202 (2.5)	1/202 (0.5)	0/165 (0)
Lee *et al*.^60^	2012	499	28.9	474/499 (95.0)		37/499 (7.4)		0/71 (0)
Shono *et al*.^61^	2011	137	29.2	122/137 (89.1)	117/137 (85.4)	5/137 (3.6)	5/137 (3.6)	5/132 (3.8)
Kim *et al*.^62^	2011	108	27.6		85/108 (78.7)	22/108 (20.4)		
Probst *et al*.^63^	2012	76	45.9	62/76 (81.6)	53/76 (69.7)	1/76 (1.3)	6/76 (7.9)	6/65 (9.2)

† For an institution that published several reports, the latest report was selected.

ESD, endoscopic submucosal dissection.

Outcomes of colorectal ESD by a summary of previous reports from multicenter studies are shown in Table 4.^44^,^64^–^68^ Although these reports included data from the early period to the more recent period of colorectal ESD without considering the learning curve, en bloc resection (endoscopic) and complete en bloc resection (histological) rates were 88.8% and 83.8% by Saito *et al*. and Tanaka *et al*., respectively. In another study, Fargat *et al*. reported that en bloc resection (endoscopic) and complete en bloc resection (histological) rates were 67.1% and 62.4%, respectively. The perforation rate was 3.3–14.0% and the delayed perforation rate was 0.4–0.7%. Postoperative bleeding occurred in 1.5–2.1% of cases.

**Table 4:** Overall data of outcomes of colorectal ESD by summary of previous multicenter study reports

Author	Year	No. institutions	No. cases	En bloc resection rate	Complete en bloc resection rate	Complications		

						Perforation rate	Delayed perforation rate	Post-ESD bleeding rate
Tsuda^64^	2006	19	1367			5.4%	0.6%	2.1%
Taku *et al*.^65^	2007	4	43			14.0%		
Tanaka *et al*.^44^	2010	194	8303		83.8%	4.8%	0.7%	1.6%
Saito *et al*.^66^	2010	10	1111	88.8%		4.9%	0.4%	1.5%
Oka *et al*.^67^	2010	39	688			3.3%		1.7%
Fargat *et al*.^68^	2011	16	85	67.1%	62.4%		36% †	

† Perforation + bleeding.

ESD, endoscopic submucosal dissection.

## Discussion

With the development of various new tools and peripheral devices and the accumulation of experience and expertise in ESD, colorectal ESD is gradually coming into widespread use in Japan.^5^,^69^ In Japan, colorectal ESD was approved for health insurance coverage in April 2012. According to a literature survey, the safety and efficacy of colorectal ESD has almost been established.^5^ Most instances of perforation that occur during colorectal ESD are usually microperforation, which can be closed completely by clipping, and a CO_2_ supply unit is used during the procedure. Hence, cases in which additional surgery is required because of perforation are very rare.^5^,^70^–^75^ Technical difficulties associated with colorectal ESD have been significantly reduced, and this procedure is growing in popularity among experienced endoscopy specialists.^76^–^81^ Nevertheless, colorectal ESD remains technically more difficult than esophageal and gastric ESD for the following reasons: (i) the anatomical features of the large intestine, a long luminal organ with many folds and flexions, mean that the endoscope cannot be well manipulated for some lesions; (ii) the intestinal wall is thin and easy to perforate; and (iii) peritonitis may occur if stool in the large intestine leaks through a perforation into the abdominal cavity. Moreover, the operator's skill varies widely, and it is therefore difficult to ensure that colorectal ESD is widely used by endoscopists. In fact, average therapeutic outcomes of ESD reported in the literature suggest that its perforation rate is higher than that achieved with EMR, and approximately 2% of ESD procedures resulted in piecemeal resection, regardless of the initial objective of complete en bloc excision. In addition, colorectal ESD places a burden on medical institutions and the economy in terms of the time required, cost, and humanpower, which seems excessive for a technique to treat benign adenomatous lesions.

In contrast, piecemeal EMR has proven to be effective mostly in adenomatous lesions, because tumors can be radically cured as long as the indications are appropriately selected. As discussed above, it is still important in clinical practice to discriminate between indications for EMR (piecemeal EMR) and those for ESD by carrying out a precise examination before treatment to determine whether the lesion is an adenoma or a carcinoma, and whether the lesion needs en bloc excision. Recently developed IEE (e.g. NBI and FICE)^12^–^15^ is currently used in clinical practice in addition to pit pattern diagnosis^6^,^9^ and facilitates precise preoperative diagnosis of colorectal tumors. Although, at present, pit pattern diagnosis or IEE with magnifying colonoscopy has not been commonly used in Western countries, recent advances in technology have introduced a new high-resolution Dual Focus Videoendoscopic System with electronic zoom function (EVIS LUCERA ELITE & EVIS EXERA III; Olympus, Tokyo, Japan), which are very simple and easy to use even by novices of magnifying colonoscopy. Without a doubt, pit pattern diagnosis or IEE with magnifying colonoscopy will become a common procedure worldwide.

In daily practice, using magnifying colonoscopy, adenoma, carcinoma in adenoma, and carcinoma without adenomatous components are well discriminated and the invasion depth of carcinoma can be determined with high precision. In the case of carcinoma in adenoma, the carcinomatous portion and adenomatous lesions can also be identified precisely. In the absence of these specific clinical backgrounds, prearranged piecemeal EMR would not be applied to adenoma or carcinoma in adenoma. However, previous studies have shown that when the number of fractions obtained in piecemeal resection increases, the rate of local recurrence of carcinoma also significantly increases.^7^ Thus, piecemeal EMR with too many fractions should be avoided because complete retrieval of the specimen might not be achieved, making a precise histopathological diagnosis more difficult. Piecemeal EMR should be planned on the basis of clinicopathological findings before treatment, and the number of fractions obtained in piecemeal resection should be minimized.^7^

In selecting a therapeutic method for colorectal adenoma or early carcinoma, detailed clinicopathological characterization of the lesion by precise preoperative diagnosis and consideration of the experience and skills of the clinicians who will carry out the procedure is important. It is also important to balance the need for completeness of cure with clinical simplicity, safety, and economical efficiency. The standardization of colorectal ESD techniques and training are important goals for the future. However, not all colonoscopists will need to carry out colorectal ESD, because ESD is indicated for less than 10% of the lesions of colon adenoma and early cancer,^82^ and there are extremely few applicable cases for the colon compared with the stomach and the esophagus.^63^ If colorectal ESD is intensively carried out by well-experienced and trained colonoscopists in a central hospital in each area, effective treatment can be attained and the experience and techniques of the procedure will be maintained and improved.

## Conclusion

Although en bloc excision is desirable for tumorous lesions if applicable, the objective of endoscopic treatment for adenomas is elimination of the lesion; however, complete en bloc excision is required for total excisional biopsy of carcinomatous lesions. Hence, even piecemeal EMR works well enough to eliminate an obvious adenoma. A time-consuming and costly treatment such as ESD should not be applied to lesions with inadequate preoperative diagnosis. Deliberately prearranged piecemeal EMR is a well-accepted treatment for adenomatous LST, aimed at achieving a localized radical cure. However, inappropriate piecemeal EMR with too many fractions must be avoided. Endoscopists who are only prepared to use piecemeal EMR with many fractions for treating a large lesion may be inexperienced in ordinary en bloc EMR and should be trained appropriately. In endoscopic treatment, we should strive for ‘clinical simplicity, economic efficiency, and safety’ as long as completeness of cure is assured. However, colorectal ESD with its current technical limitations cannot as yet be recommended as a localized radical cure as it does not meet the objective of ‘clinical simplicity, economic efficiency, and safety’. In the event that endoscopists are not confident whether the preoperative diagnosis allows for selecting the correct treatment, they should either undergo additional training or refer the patient to a specialized institution. Clinical research on the ESD technique and ordinary clinical practice using ESD must be considered separately, and endoscopists should not carry out superfluous ESD merely for their own curiosity or research interest. EMR may go out of use when colorectal ESD can be carried out more simply, safely, and inexpensively, but the ESD technique does not conform to these requirements at present. However, in the near future, we anticipate further significant progress on a parallel with the establishment of grounded education and a training system.
